# A retrospective analysis of EBV‐DNA status with the prognosis of lymphoma

**DOI:** 10.1111/jcmm.17543

**Published:** 2022-09-06

**Authors:** Lihua Qiu, Junqi Si, Junnan Kang, Zehui Chen, Rexidan Nuermaimaiti, Zhengzi Qian, Lanfang Li, Shiyong Zhou, Mingjian James You, Huilai Zhang, Chen Tian

**Affiliations:** ^1^ Tianjin Medical University Cancer Institute and Hospital, National Clinical Research Center for Cancer, Key Laboratory of Cancer Prevention and Therapy, Tianjin's Clinical Research Center for Cancer Tianjin China; ^2^ Hotan District People's Hospital Hotan China; ^3^ Department of Hematopathology The University of Texas MD Anderson Cancer Center Houston Texas USA

**Keywords:** EBV‐DNA, hemophagocytic syndrome, lymphoma, prognosis

## Abstract

Epstein–Barr virus (EBV) infection is proved to be associated with clinicopathology of lymphoma. However, little is known about the relationship between EBV‐DNA status after treatment and prognosis. In this study, real‐time polymerase chain reaction (PCR) was used for quantitative detection of EBV‐DNA load in peripheral blood of all 26,527 patients with lymphoma, and the clinical characteristics and prognosis of 202 patients were retrospectively analysed, including 100 patients with positive EBV‐DNA and 102 randomly selected patients with negative EBV‐DNA. We found that the average rate of EBV‐DNA positivity in lymphomas was 0.376%, and EBV‐DNA‐positive patients presented higher risk with elevated lactate dehydrogenase (LDH) and β2‐MG level, B symptoms, secondary hemophagocytic syndrome and lower objective response rate compared to EBV‐DNA‐negative patients. Multivariate analysis revealed EBV‐DNA‐positive patients had inferior progression‐free survival (PFS) and overall survival (OS) and EBV‐DNA level before treatment was related to PFS but not OS of T/NK cell lymphoma. In T/NK cell lymphoma, EBV‐DNA converting negative after treatment was correlated with better PFS but not OS, and second‐line therapy could induce more EBV‐DNA‐negative conversion compared to CHOP‐based therapy. In all, EBV‐DNA positivity before treatment can be a biomarker representing the tumour burden and an independent prognostic factor. EBV‐DNA‐negative conversion after treatment is a good prognostic factor for T/NK cell lymphomas.

**Novelty statements**

1. EBV‐DNA‐positive patients presented high risk with elevated LDH and β2‐MG level, B symptoms, secondary hemophagocytic syndrome, and low objective response rate.
2. EBV‐DNA‐positive patients had inferior progression‐free survival and overall survival.
3. High EBV‐DNA level before treatment was correlated with poor PFS of T/NK cell lymphoma.
4. EBV‐DNA‐negative conversion after treatment was a good prognostic factor for T/ NK cell lymphomas.
5. Second‐line therapy resulted in higher EBV‐DNA‐negative conversion rate of T/NK cell lymphomas compared to CHOP regimen.

## 1 INTRODUCTION

Epstein–Barr virus (EBV), a ubiquitous human herpes virus characterized by an asymptomatic latency after primary infection,^1^ is implicated in the development of various haematological diseases, including Hodgkin lymphoma (HL),^2^ non‐Hodgkin lymphoma (NHL),^3^ and post transplantation lymphoproliferative disorder (PTLD).^4^ EBV‐associated lymphomas can be divided into those occurring in immunodeficient individuals, which are true virally driven lymphomas, such as PTLD and HIV‐associated immunoblastic lymphoma, and those occurring in immunocompetent individuals. The latter group includes Burkitt lymphoma, Hodgkin's lymphoma, diffuse large B‐cell lymphoma, extranodal NK/T cell lymphoma nasal type, and so on^5^ and EBV infection is a cofactor rather than the driving influence.

Epstein–Barr virus spreads through oral cavity, proliferates in the throat, and then lurks in B lymphocytes, usually presenting as a latent infection without clinical symptoms. Given the long‐term latent infection, EBV antibody detection is insufficient to diagnose EBV‐associated lymphomas.^6^ Epstein Barr encoded RNA (EBER) and EBV‐DNA are used to define reactivation of EBV. EBER in situ hybridization (FISH) assay is considered to be the gold standard for the detection and diagnosis of EBV active infection.^7^ However, biopsies are usually not performed when tumour biopsy tissue is difficult to obtain or when the patient is refractory or relapsed lymphoma.^8^ Detection and quantification of EBV nucleic acids in peripheral blood by polymerase chain reaction (PCR) has also been widely used in the diagnosis and monitoring of EBV‐associated lymphomas.^9^ However, whether EBV‐DNA conversion to negative after treatment and qualitative results of EBV‐DNA level before treatment could affect prognosis are unclear. To elucidate these questions, we analysed the clinical characteristics and prognosis of immunocompetent patients with EBV associated lymphoma.

HLH is a rapidly progressive disease with a high fatality rate, which can occur secondary to lymphoma or severe pathogen infection, and its main clinical manifestations are persistent fever, hepatosplenomegaly, and pancytopenia. Previous studies showed that EBV‐associated HLH was the most prevalent subtype.^10^ In order to study the relationship between EBV active infection and the development of HLH in lymphoma patients, we also analysed the clinical characteristics and prognosis of EBV‐associated lymphoma patients with HLH.

## 2 MATERIALS AND METHODS

### 2.1 Patients

The positivity rates of EBV‐DNA in peripheral blood of 26,527 patients who were definitively diagnosed as lymphoma between May 1, 2010 and May 1, 2021 in Department of Lymphoma at Tianjin Medical University Cancer Institute and Hospital were calculated. And the clinical characteristics and prognosis of 202 patients were retrospectively analysed, including 100 EBV‐DNA‐positive patients and 102 randomly selected EBV‐DNA‐negative patients. Equidistant random sampling was used to number the patients according to the time of admission. Since the total number of patients is more than 26,000 and there are 100 EBV‐positive patients, the sampling interval is set at 260. Select a random number as the sampling unit in the first sampling interval and conduct equidistant sampling according to the sampling interval. A total of 102 negative patients were sampled and statistically analysed. This study and all experiment protocols were approved by the Research Ethics Committee of Tianjin Medical University Cancer Institute and Hospital and performed in accordance with relevant guidelines and regulations. Informed consent of all patients was obtained.

### 2.2 Treatment

The first‐line chemotherapy regimen was based on cyclophosphamide, doxorubicin, vincristine and prednisone (CHOP). The second‐line regimens include: DHAP (dexamethasone, cisplatin and cytarabine), DA‐EPOCH (cyclophosphamide, etoposide, vincristine, doxorubicin and prednisone), GDP (gemcitabine, cisplatin, dexamethasone), Gemox (gemcitabine, Oxaliplatin), and ICE (Ifosfamide, carboplatin, etoposide). All patients were followed up to the date of death or May 1, 2021 (median follow‐up: 23.92 months), with 37 patients (29 EBV‐DNA‐positive patients and 8 EBV‐DNA‐negative patients) lost follow‐up.

### 2.3 Clinical evaluation index

The response evaluation was divided into complete response (CR), partial response (PR), stable disease (SD), and progressive disease (PD) according to the 2007 Revised International Working Group Response Criteria for Malignant Lymphoma. OS was measured from the time of diagnosis to the date of death or the final follow‐up. PFS was measured from the time of diagnosis to the date of disease progression, death, or the final follow‐up.

### 2.4 EBV‐DNA quantification

Real‐time polymerase chain reaction (PCR) was used to detect EBV‐DNA in peripheral blood. DNA was extracted from 150 μl plasma using Kit Ribo Virus. Amplification was performed with EBV Real‐TM Quant following the standard manufacturer's instructions in reaction volumes of 25 μl and using the Quant Studio Dx Real‐Time PCR Instrument. The primers of the latent membrane protein (LMP2) region of EBV‐DNA were as follows: forward: 5′‐AGC TGT AAC TGT GGT TTC CAT GAC‐3′; reserve: 5′‐GCC CCC TGG CGA AGA G‐3′. 5 × 10^2^ copy/ml was defined to be the critical value. Higher than 5 × 10^2^ copy/ml was considered to be EBV‐DNA positive. EBV‐DNA load in peripheral blood was measured before initial treatment and after four cycles of chemotherapy.

### 2.5 Statistical analysis

Univariate analysis was performed using the Kaplan–Meier model, multivariate analysis was performed using the Cox regression model, and the differences were assessed using the log‐rank test. The qualitative data were compared using the χ^2^ test. *p* < 0.05 was considered to be statistically significant different. All statistical analyses were performed using SPSS 26.0 software.

## 3 RESULTS

### 3.1 Proportion of EBV‐DNA positivity in each subtype

Among all lymphoma cases, 100 patients were EBV‐DNA positive, with a total EBV active infection rate of 0.376%. The active infection rate of EBV in HL was 0.196%. Among aggressive B‐cell lymphoma, EBV active infection rate of B‐cell lymphoblastic lymphoma (B‐LBL) was higher than that of DLBCL (0.358% vs. 0.264%). For indolent B‐cell lymphoma, chronic lymphocytic leukaemia (CLL) was the most common subtype infected with EBV. The active infection rate of EBV in T/NK cell lymphoma was higher compared to B cell lymphoma, among which angioimmunoblastic T‐cell lymphoma (AITL) is the highest (3.56%), followed by peripheral T cell lymphoma (PTCL). Taken together, T/NK cell lymphomas were more commonly associated with EBV‐DNA active infection (Table 1).

**TABLE 1:** Proportion of EBV positive lymphoma in each subtype

Subtype		Total (*n*)	EBV positive (*n*)	Infection rate (%)
Aggressive B‐cell lymphoma	DLBCL	10,976	29	0.264
	B‐LBL	279	1	0.358
Indolent B‐cell lymphoma	FL	5031	3	0.059
	MZL	641	1	0.156
	SLL/CLL	314	1	0.318
T or NK/T cell lymphoma	NK/T	2744	30	1.093
	ALCL	942	2	0.212
	AITL	337	12	3.56
	PTCL	497	11	2.21
	T‐LBL	192	1	0.521
HL		4574	9	0.196
Total		26,527	100	0.376

### 3.2 Clinical characteristics between EBV‐DNA‐positive and ‐negative patients

The patients' characteristics are listed in Table 2. The total number of EBV‐DNA‐positive patients in our large sample over 1 years is 100. One hundred and two patients with negative EBV‐DNA were randomly sampled as controls. Of the 202 patients, 130 (64.4%) were male and 72 (35.6%) were female. The average age was 52.56 ± 15.77 years old, and the median age was 51 years old (range 18–83 years). Seventy‐six patients (37.6%) were older than 60 years, 139 patients (68.8%) presented with Ann Arbor stage III‐IV, and 99 patients (50%) had elevated LDH levels (>250 U/L). Nearly half of the patients (48.8%) had normal β2‐MG level (≤2.6 mg/L), 74.0% patients were categorized into the low to intermediate risk group (IPI ≦ 2) according to the International Prognostic Index (IPI). B symptoms (57.0% vs. 26.5%, *p* < 0.001) and hemophagocytic syndrome (13.0 vs. 0.0%, *p* < 0.001) were more common in EBV‐DNA‐positive patients. EBV‐DNA‐positive patients obtained higher LDH level (66.7% vs. 34.3%, *p* < 0.001), more advanced Ann Arbor stage (78.0% vs. 59.8%, *p* = 0.005), higher β2‐MG (67.7% vs. 35.3%, *p* < 0.001), and higher IPI score (32.3% vs 19.8%, *p* = 0.044) compared to EBV‐DNA‐negative patients. However, the involvement of liver, spleen, bone, and the lymph node size between these two groups showed no significant difference. EBV‐DNA‐negative patients had a significantly higher ORR (74.5% vs. 57.0%, *p* = 0.009) compared to positive patients.

**TABLE 2:** Baseline characteristics of EBV‐DNA‐positive and ‐negative patients

Factors		EBV‐DNA positive	EBV‐DNA negative	Total *n* (%)	χ^2^	*p* value
Sex	Male	68	62	130 (64.4)	1.146	0.284
	Female	32	40	72 (35.6)		
Age	≥60	41	35	76 (37.6)	0.962	0.327
	<60	59	67	126 (62.4)		
Ann Arbor stage	I‐II	22	41	63 (31.2)	7.790	0.005
	III‐IV	78	61	139 (68.8)		
Disease status after treatment	PR/CR	57	76	133 (65.8)	6.884	0.009
	PD/SD	43	26	69 (34.2)		
B symptom	Yes	57	27	84 (41.6)	19.347	<0.001
	No	43	75	118 (58.4)		
LDH level	>250	64	35	99 (50)	20.706	<0.001
	≤250	32	67	99 (50)		
β2‐MG	≤2.6	32	66	98 (48.8)	21.086	<0.001
	>2.6	67	36	103 (51.2)		
IPI	≤2	67	81	148 (74)	4.074	0.044
	>2	32	20	52 (26)		
Liver involvement	Yes	2	7	9 (4.5)	2.754	0.097
	No	97	95	192 (95.5)		
Spleen involvement	Yes	43	42	85 (42.3)	0.105	0.746
	No	56	60	116 (57.7)		
Lymph node	≥7 cm	6	6	12 (5.9)	0.001	0.972
	<7 cm	94	96	190 (94.1)		
Bone marrow involvement	Yes	17	10	27 (13.4)	3.470	0.176
	No	81	92	173 (86.1)		
Hemophagocytic syndrome	Yes	13	0	13 (6.4)	14.172	<0.001
	No	87	102	189 (93.6)		

**FIGURE 1:**
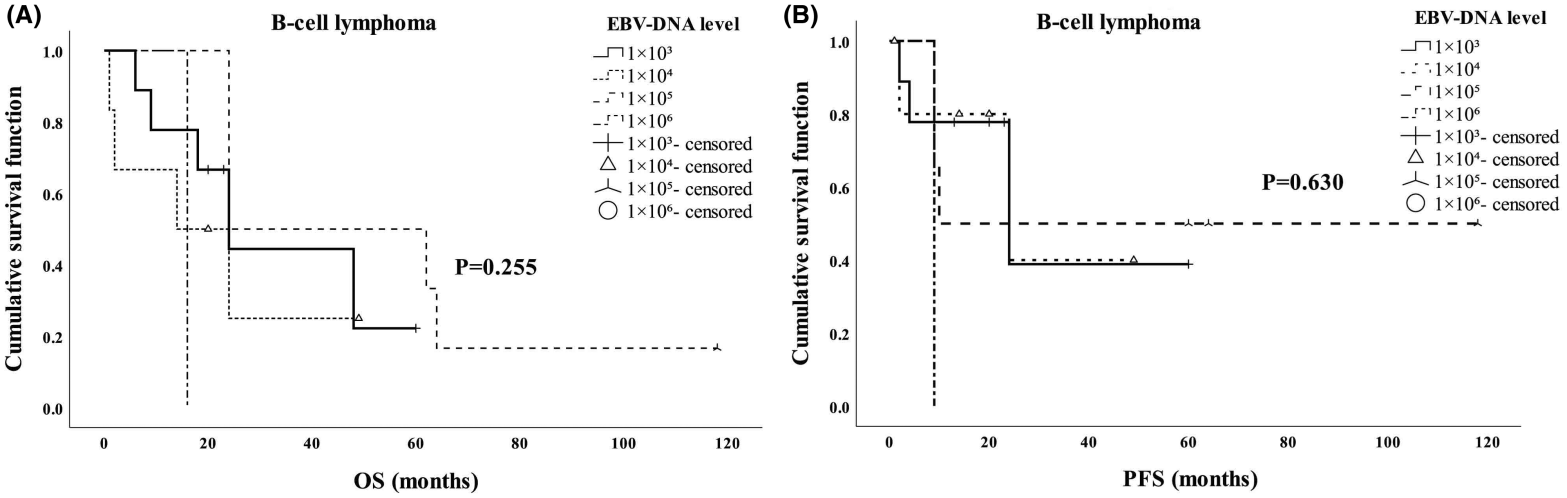
The EBV‐DNA level in peripheral blood to the prognosis of B cell lymphoma. (A) EBV‐DNA level before treatment showed no relationship with the OS of B cell lymphoma. (B) EBV‐DNA level before treatment was not related to PFS of B‐cell lymphoma

**FIGURE 2:**
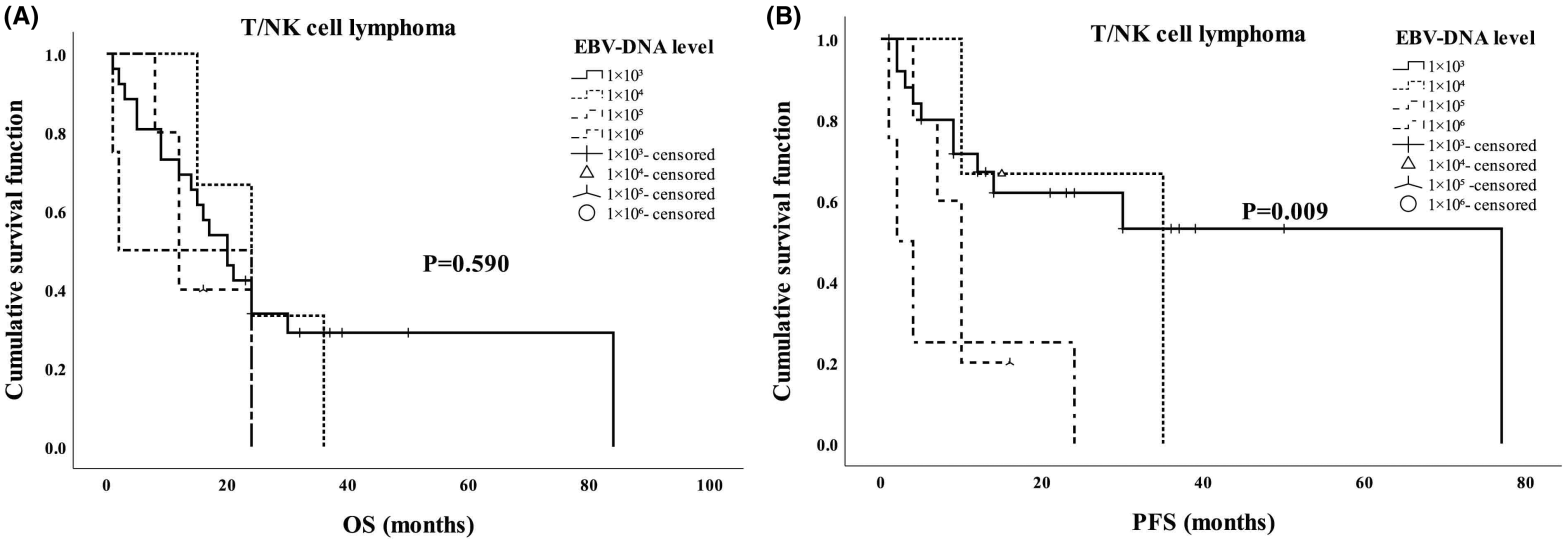
The EBV‐DNA level in peripheral blood to the prognosis of B cell lymphoma. (A) EBV‐DNA level before treatment was not related to OS of T/NK‐cell lymphoma. (B) High EBV‐DNA level before treatment predicted poor PFS of T/NK‐cell lymphoma

In order to investigate whether the level of EBV‐DNA before treatment affect the prognosis of EBV‐positive patients, the survival time of patients with EBV‐DNA levels of 1 × 10^3^, 1 × 10^4^, 1 × 10^5^ and 1 × 10^6^ was compared. The results showed that EBV‐DNA level before treatment could affect PFS of patients with T/NK cell lymphoma (Figure 2B, *p* = 0.009), but had no effect on OS and PFS of patients with B cell lymphoma (Figure 1) nor the OS of patients with T/NK cell lymphoma (Figure 2A).

### 3.3 Different EBV‐DNA status after treatment

**FIGURE 3:**
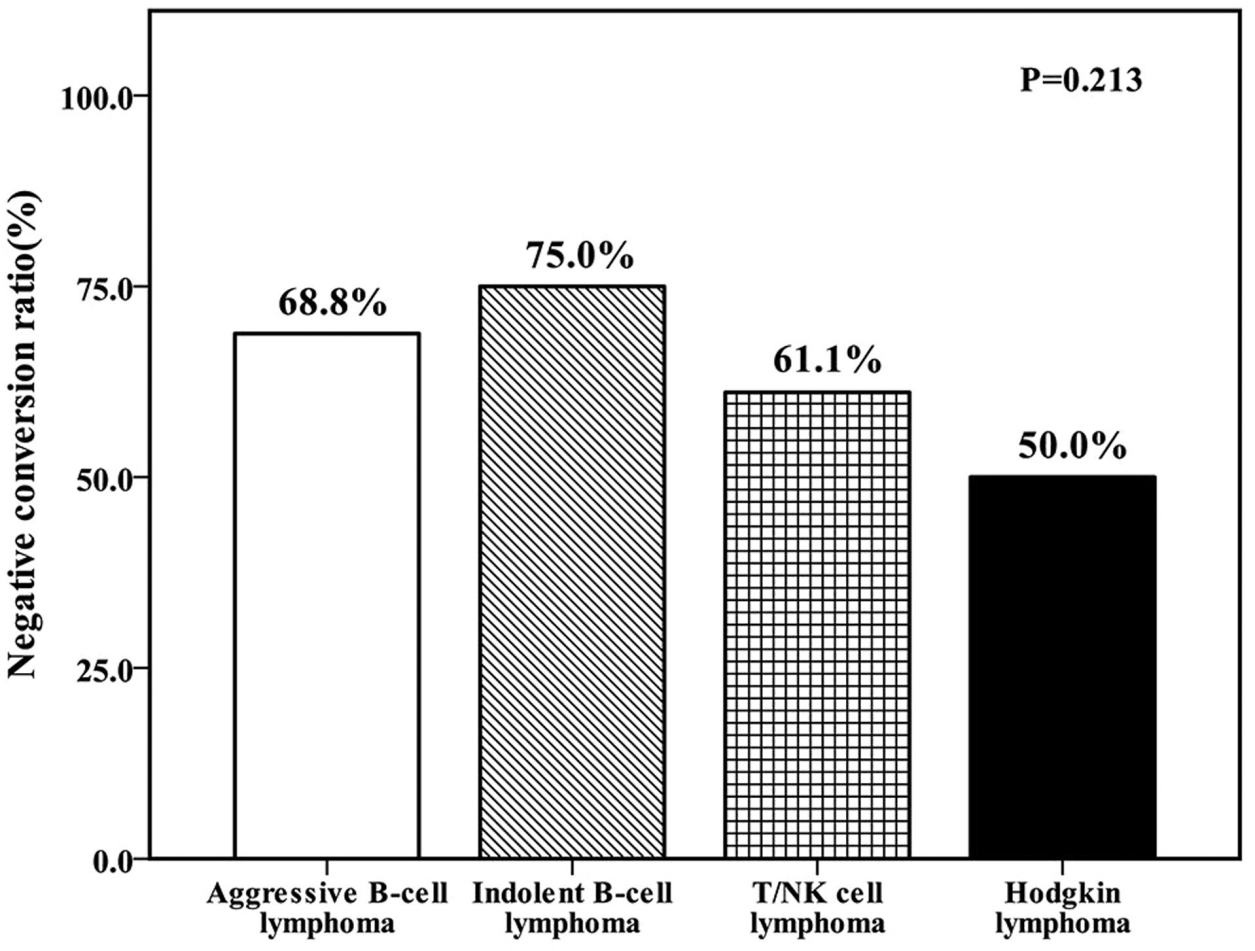
The EBV‐DNA‐negative conversion rate of different lymphoma types after treatment

EBV‐DNA status in patients' peripheral blood was re‐detected after four cycles of treatment. The negative conversion rates of different subtypes of lymphomas were shown in Figure 3, in which indolent B‐cell lymphoma is higher than other subtypes. Kaplan–Meier analysis revealed that EBV‐DNA converting negative after treatment was correlated with improved PFS of T/NK cell lymphoma (Table 3, *p* = 0.001) but had no effect on OS (Table 3, *p* = 0.226). For B‐cell lymphoma, there was no significant difference in EBV‐DNA‐negative conversion rate between first‐line CHOP regimen and second‐line therapy (*p* = 0.226, Table 3). However, for T/NK cell lymphoma, second‐line therapy appeared to result in higher EBV‐DNA‐negative conversion rate compared to CHOP‐based therapy (*p* = 0.009, Table 3).

**TABLE 3:** Analysis of different EBV‐DNA status after treatment

Factors			EBV‐DNA turn negative after treatment	EBV‐DNA still positive after treatment	Total *n* (%)	χ^2^	*p* value
OS	B‐cell lymphoma		8	3	11 (28.2)	0.279	0.598
	T or NK/T cell lymphoma		16	12	28 (71.8)	1.004	0.316
PFS	B‐cell lymphoma		8	3	11 (28.2)	0.596	0.440
	T or NK/T cell lymphoma		16	12	28 (71.8)	7.094	0.008
LDH	High		29	16	45 (70.3)	0.245	0.621
	Normal		11	8	19 (29.7)		
Therapy	B‐cell lymphoma	CHOP‐based	12	3	15 (26.7)	1.466	0.226
		Second‐line	2	2	4 (7.2)		
	T or NK/T cell lymphoma	CHOP‐based	3	8	11 (19.7)	6.782	0.009
		Second‐line	19	7	25 (46.4)		

### 3.4 Prognostic analysis

The univariate analysis showed that EBV‐DNA, age, response status, β2‐MG level, LDH level, and hemophagocytic lymphohistiocytosis (HLH) were prognostic factors relating to OS and PFS (*p* < 0.05), while patients with IPI score >2 were associated with poor OS (*p* = 0.013) and patients with B symptoms had worse PFS (*p* = 0.026). Multivariate analysis found that age, EBV‐DNA, response status, and HLH were significantly independent prognostic factors for both poor PFS and OS (*p* < 0.05, Table 4).

**TABLE 4:** Univariate analysis and multivariate analysis of factors potentially associated with survivals

Factors	OS			PFS		
	Univariate analysis	Multivariate analysis		Univariate analysis	Multivariate analysis	
		HR (95% CI)	*p* value		HR (95% CI)	*p* value
initial EBV‐DNA	*p* < 0.001	0.542 (0.326–0.902)	0.018	*p* < 0.001	0.461 (0.237–0.897)	0.022
Age	*p* < 0.001	2.131 (1.280–3.548)	0.004	*p* < 0.001	2.318 (1.214–4.425)	0.011
Sex	0.615			0.612		
Ann Arbor stage	0.100	0.866 (0.477–1.570)	0.635	0.057	1.131 (0.543–2.393)	0.748
Disease status after treatment	*p* < 0.001	2.595 (1.640–4.140)	<0.001	*p* < 0.001	4.571 (2.466–8.473)	<0.001
B symptom	0.070	0.805 (0.505–1.284)	0.363	0.026	0.663 (0.362–1.216)	0.184
β2‐MG	0.001	1.160 (0.682–1.970)	0.584	0.008	1.001 (0.510–1.964)	0.998
IPI	0.013	0.980 (0.550–1.747)	0.947	0.234	0.831 (0.408–1.694)	0.611
LDH level	0.001	0.709 (0.426–1.182)	0.188	0.010	0.957 (0.497–1.845)	0.896
Liver involvement	0.796			0.213		
Spleen involvement	0.894			0.852		
Bone marrow involvement	0.724			0.698		
Lymph node	0.616			0.841		
Hemophagocytic syndrome	*p* < 0.001	0.906 (0.437–1.878)	0.791	*p* < 0.001	0.633 (0.278–1.440)	0.275

### 3.5 EBV active infection was related to secondary HLH

Among the 26,527 recruited lymphoma patients, a total of thirteen patients were accompanied with HLH. EBV‐DNA was positive in all HLH patients, indicating that EBV active infection was related to the occurrence of HLH (*p* < 0.001, Table 2). Univariate and multivariate analyses indicated that HLH was an independent factor affecting the prognosis (Table 4). In addition, we found that the HLH patients had higher levels of LDH (*p* = 0.045), β2 MG (*p* < 0.001), and EBV‐DNA (*p* < 0.001) compared to those without HLH. The average value of EBV‐DNA in patients with HLH (36 × 10^5^ copy/ml) was significantly higher than that in EBV‐positive patients without HLH (6 × 10^5^ copy/ml) (*p* < 0.001).

## 4 DISCUSSION

Epstein–Barr virus, a member of the human herpesvirus family, is widespread in world's population^11^ and is carried as a latent asymptomatic infection in individuals.^12^ Persistent EBV active infection is considered a high‐risk factor for nasopharyngeal carcinoma and malignant lymphomas, including Hodgkin and non‐Hodgkin lymphomas.^5^ B cells are known to be the primary lymphoid target of EBV infection. EBV‐associated NK and T cell lymphoproliferative diseases are more common in Asia.^13^ A study in China indicated that EBV‐DNA was more frequently detected in T/NK cell lymphomas than in B cell lymphomas.^14^ A similar result was observed in our study, and EBV‐DNA positivity rate was higher in T/NK cell lymphomas than that in B‐cell lymphomas.

Some studies revealed that EBV‐DNA positivity before treatment could reflect the tumour burden.^15^, ^16^, ^17^, ^18^, ^19^ We found that EBV‐DNA‐positive patients generally presented more risk factors such as elevated LDH level, higher β2‐MG level, and B symptoms compared to EBV‐DNA‐negative patients. In addition, patients with positive EBV‐DNA are more likely to develop HLH. These data suggest that the EBV‐DNA positivity may be used as a surrogate biomarker for assessing tumour burden.

Clinically, EBV‐DNA positivity is a useful prognostic biomarker in EBV‐associated lymphomas. Liang et al^7^ showed that the pretherapy EBV‐DNA positivity is a better biomarker for poor OS than EBER. Our multivariate analysis also revealed that EBV‐DNA positivity was an independent prognostic factor. However, no reports on EBV‐DNA level to the prognosis of lymphoma patients were published as we known. We found that EBV‐DNA level before treatment could affect PFS of patients with T/NK cell lymphoma, but had no effect on OS and PFS of patients with B cell lymphoma nor the OS of patients with T/NK cell lymphoma.

Previous studies reported that EBV‐DNA status after treatment was correlated with the treatment response and survival of T/NK cell lymphoma patients.^20^, ^21^ Similar results were observed in our study. We divided lymphomas into B‐, T/NK‐lineages and HL to investigate whether the change of EBV‐DNA status after treatment could affect the survival. In T/NK cell lymphomas, patients with negative EBV‐DNA conversion showed a better PFS than patients who remained positive. However, negative EBV‐DNA conversion did not significantly affect the OS and PFS of B‐cell lymphoma and HL patients. We also found that in patients with EBV‐DNA‐positive T/NK lymphoma, second‐line treatment seems to lead to a high EBV‐DNA‐negative conversion rate. In addition, we found that the change of LDH level after treatment was positively correlated with EBV‐DNA, suggesting that the change of EBV‐DNA status could affect LDH level.

HLH is a rapidly progressive, highly fatal disease that may occur either as a result of the lymphoma disease itself or pathogen infection during immunosuppression.^22^ Secondary HLH is often associated with a variety of underlying diseases, such as infection, tumour, and rheumatic diseases. Among the infectious factors, EBV infection is the most important one. We found that HLH patients in the study were all EBV‐DNA positive, indicating that EBV active infection increased the risk of HLH in lymphoma patients. Our study also showed that the prognosis of HLH patients was significantly worse, which may be related to the high copy number of EBV‐DNA in lymphoma patients.

In our study, we found that EBV active infection rate in T/NK cell lymphoma was significantly higher than that in B cell lymphoma, suggesting that EBV may play an important effect in the pathogenesis of T/NK cell lymphoma. Our results also revealed that EBV‐DNA positivity before treatment could be a surrogate biomarker representing the tumour burden and was correlated to poor prognosis of lymphoma patients. EBV‐DNA level before treatment was an independent prognostic factor for T/NK cell lymphoma. EBV‐DNA turning negative after treatment was related to improved PFS of T/NK cell lymphoma, and second‐line therapy resulted in increased EBV‐DNA‐negative conversion.

## AUTHOR CONTRIBUTIONS

**Lihua Qiu:** Data curation (equal); writing – original draft (equal). **Junqi Si:** Data curation (equal); writing – original draft (equal). **Junnan Kang:** Data curation (equal); writing – original draft (equal). **Zehui Chen:** Writing – review and editing (equal). **Rexidan Nuermaimaiti:** Methodology (equal); resources (equal). **Zhengzi Qian:** Data curation (equal). **Lanfang Li:** Data curation (equal). **Shiyong Zhou:** Data curation (equal). **Mingjian James You:** Methodology (equal). **Huilai Zhang:** Resources (equal); writing – review and editing (equal). **Chen Tian:** Formal analysis (equal); funding acquisition (equal); methodology (equal); validation (equal); writing – review and editing (equal).

## FUNDING INFORMATION

C.T. is supported by Grant ZC20171 from Tianjin Health Science and Technology Project and Natural Science Foundation of Xinjiang Autonomous Region (2022D01A09).

## CONFLICT OF INTEREST

The authors declare that they have no conflicts of interest.

## Data Availability

The data sets used and/or analysed during the current study are available from the corresponding author on reasonable request.
